# Evaluation of care with intravitreal aflibercept treatment for UK patients with diabetic macular oedema: DRAKO study 24-month real-world outcomes

**DOI:** 10.1038/s41433-023-02409-y

**Published:** 2023-03-20

**Authors:** Sobha Sivaprasad, Faruque Ghanchi, Simon P. Kelly, Ajay Kotagiri, James Talks, Peter Scanlon, Hellen McGoey, Andrew Nolan, Moneeb Saddiq, Jackie Napier

**Affiliations:** 1 National Institute for Health Research, Moorfields Biomedical Research Centre, London, UK; 2 Bradford Teaching Hospitals NHS Foundation Trust, Bradford, UK; 3 Bolton Hospital NHS Foundation Trust, Bolton, UK; 4 South Tyneside and Sunderland NHS Foundation Trust, Sunderland, UK; 5 Newcastle Upon Tyne Hospitals NHS Foundation Trust, Newcastle Upon Tyne, UK; 6 Gloucestershire Hospitals NHS Foundation Trust, Cheltenham, UK; 7 Bayer Plc, Reading, UK; 8 Ipsen UK Ltd, Slough, UK; 9 O4 Research Limited, Belfast, UK

**Keywords:** Retinal diseases, Health care

## Abstract

**Background/ Objectives:**

DRAKO (NCT02850263) was a 24-month, prospective, observational, multi-centre cohort study that enrolled patients diagnosed with diabetic macular oedema (DMO) including central involvement. The study aimed to evaluate standard of care intravitreal aflibercept (IVT-AFL) treatment in the UK. This analysis describes the 12-month outcomes for patients with prior anti–vascular endothelial growth factor (VEGF) treatment for DMO other than IVT-AFL (C2), and 2-year outcomes for both anti-VEGF treatment–naïve patients (C1) and C2 patients.

**Methods:**

Study eyes were treated with IVT-AFL as per local standard of care. Mean changes in best-corrected visual acuity (BCVA) in ETDRS letters and central subfield thickness (CST) were stratified by baseline factors. Changes in diabetic retinopathy assessments, glycated haemoglobin A_1c_ levels and vision-related quality of life (QoL) were evaluated alongside numbers of injections administered and safety outcomes.

**Results:**

For C1, mean (SD) changes from baseline in BCVA of +0.7 (12.7) letters and CST of –123.3 (104.3) µm were observed at Month 24. For C2, mean (SD) changes from baseline for BCVA of + 0.2 (10.2) letters and –0.3 (13.0) letters, and CST of –79.1 (137.6) µm and −91.6 (132.9) µm, were observed at 12 and 24 months, respectively. In Year 2, C1 and C2 patients received a mean of 3.7 and 4.3 injections, respectively.

**Conclusions:**

Year 2 results indicate that IVT-AFL is an effective treatment for DMO in real-world UK clinical practice, despite relatively low injection numbers. The high baseline visual acuity and QoL scores were maintained and there was further improvement in anatomical outcomes.

## Introduction

Globally, diabetes affects more than half a billion people, and this will rise to 783.2 million in the 20–79-year-old population by 2045 [1]. Diabetic macular oedema (DMO), a microvascular complication of the disease, is the most common cause of visual acuity (VA) loss in patients with diabetes, accounting for around 75% of all cases [2]. One in four patients with diabetes can expect to develop DMO in their lifetime [3–6].

Over the last decade, the use of anti–vascular endothelial growth factor (VEGF) treatments has become the first-line therapy of choice for management of vision loss from DMO.

Intravitreal aflibercept (IVT-AFL; Eylea) is an anti-VEGF treatment with an innovative fusion protein design that allows a proactive, every-other-month treatment regimen (after five initial monthly doses) with no monitoring requirement between injections. This was an important development, as monthly monitoring places a significant burden on patients, their caregivers, physicians and the wider healthcare system. After the first 12 months of treatment with IVT-AFL, the treatment interval may be extended based on visual and anatomical outcomes, further reducing the treatment burden (IVT-AFL summary of product characteristics [SmPC] recommended posology for DMO treatment) [7].

DRAKO represents the first UK-based, prospective, observational study to assess the effectiveness of standard of care IVT-AFL treatment in patients with DMO across a wide range of centres.

Although randomised control trials (RCTs) are the gold standard, providing standardisation and minimising confounders, they do not always reflect ‘real-world’ outcomes due to the difficulty that patients, caregivers and healthcare providers have in following optimum treatment regimens in the real-world setting.

Observational studies such as DRAKO are valuable as they report and evaluate outcomes based on locally defined treatment practices, outside the rigorous clinical trial setting, enabling outcome characterisation within a more representative population and treatment environment.

DRAKO enrolled 750 patients from 35 centres across the UK in two cohorts – anti-VEGF treatment–naïve or non–treatment-naive patients with prior anti-VEGF treatment for DMO other than IVT-AFL. Patients were followed for 2 years with 12-month best-corrected visual acuity (BCVA) and central subfield thickness (CST) outcomes selected as the primary endpoints, as RCT data for anti-VEGF treatments in DMO have demonstrated that most VA gains are observed in Year 1 [8, 9].

The 12-month outcomes for DRAKO in anti-VEGF treatment–naive patients (*n* = 507) were reported in 2021 [10]. Patients were diagnosed and treated with a mean (standard deviation [SD]) baseline BCVA of 71.4 (12.0), a significantly higher level than seen in RCTs. This finding indicated that the UK diabetic retinopathy screening programme is effective, with 63.1% of patients presenting with good baseline vision (BCVA ≥ 70 letters [mean 78.1]).

BCVA and CST outcomes improved from baseline, with a mean (SD) change in BCVA of + 2.5 (12.2) letters and CST of −119.1 (116.4) µm. A 7.3-letter gain was observed in patients with baseline BCVA < 70 letters and the mean number (SD) of injections in Year 1 was 6.4 (2.1).

The increase in BCVA was lower than that seen in RCTs which can be explained by the protocol-driven higher injection numbers and increased opportunity for larger BCVA gains from the lower baseline of patients participating in the RCTs.

The second year of follow-up was designed to assess whether gains in Year 1 could be maintained or improved and to capture treatment patterns beyond the defined SmPC posology period [7].

Here we describe the 12-month outcomes for the non–treatment-naive cohort and 2-year outcomes for both the anti-VEGF treatment–naive and non–treatment-naive cohorts.

## Materials and methods

### Study design

DRAKO (NCT02850263) was a 24-month, prospective, observational, multi-centre, non-comparative cohort study evaluating the effectiveness of IVT-AFL for the treatment of patients with centre-involving DMO within UK routine clinical practice. The study enrolled adult patients with a confirmed diagnosis of DMO with central involvement from 35 NHS hospitals across the UK between July 2016 and April 2018. The decision to treat with IVT-AFL was made prior to and independently of study involvement. Eligible patients were enrolled consecutively and irrespective of baseline VA.

Two patient cohorts were assessed in the study – anti-VEGF treatment–naive (*N* = 507) and non–treatment-naive (*N* = 241) cohorts. The non–treatment-naive cohort had not received anti-VEGF treatment for DMO within the previous 28 days. The study was approved by the Northwest Liverpool East Research Ethics Committee (16/NW/0238) and was conducted in accordance with the Declaration of Helsinki. All participants provided written informed consent.

Regulatory approval was not required as all treatments and assessments were conducted as per local standard-of-care procedure for DMO management, with baseline and post-baseline visits being recorded throughout the 2-year follow-up period. Refracted visual acuity was recorded at baseline and at annual time-points. For data collection purposes, the Month 12 and Month 24 visits were nominated by the site and defined as Month 12 or Month 24 ± 1 month from the patient’s baseline visit. Quality of Life (QoL) was measured via completion of a NEI VFQ-25 questionnaire at Baseline, Month 12, and Month 24.

### Outcome measures

The primary outcome measures were the mean change from baseline in Early Treatment Diabetic Retinopathy Study letters measured by BCVA and the mean change in CST as determined by spectral domain optical coherence tomography at Month 12 for both cohorts. Outcomes for the treatment-naive cohort were previously reported [10]. Here, we report the non–treatment-naive cohort primary outcomes, in addition to secondary and exploratory outcomes for both cohorts.

Secondary objectives included change from baseline in: (1) percentage gain and loss of ≥ 5, ≥ 10 or ≥ 15 letters; (2) BCVA and CST stratified by pre-defined baseline factors; (3) vision-related QoL measured using the National Eye Institute Visual Function Questionnaire 25-item version (NEI VFQ-25); (4) diabetic retinopathy (DR) score, measured using the English National Screening Committee (English) or Scottish DR Grading Scheme (Scottish) classifications dependent on site preference; and (5) glycated haemoglobin (HbA_1c_) at Months 12 and 24 for both cohorts. Exploratory analysis of the number of injections by letter gain or loss, and baseline subgroups was conducted, in addition to evaluating outcomes versus the number of injections administered in Year 2.

### Statistical analysis

Interim analysis was conducted for each cohort separately upon completion of the first year of follow-up, and Month 24 analysis was conducted upon completion of the study.

Two populations were defined for each patient cohort at Months 12 and 24: a ‘per protocol window’ (PPW) population, which included patients with BCVA or CST data available at baseline and the Month 12 or 24 visits; and a full analysis set population, which included patients with BCVA or CST available at baseline and at least one follow-up visit; missing values were imputed based on the last observation carried forward method. The PPW population is reported throughout this data summary.

The Month 24 analysis was conducted as described previously for the treatment-naive cohort at Month 12 [10]. Quantitative variables were summarised by descriptive statistics, and categorical variables using frequency distributions and percentages. Outcomes were stratified by pre-defined baseline subgroups; BCVA ( ≤ 49, 50–69, ≥ 70, < 70 letters) and CST ( < 300, 300–399, 400–499, ≥ 500 µm). No comparative tests of significance were conducted for the primary objectives, as no specific hypothesis was being tested. The association between ≥ 5, ≥ 10, and ≥ 15 BCVA letter gains or losses and the number of injections administered in Year 1 and Year 2 were assessed and correlation coefficient and p-values calculated.

Safety was assessed for all patients who provided written informed consent. Reported adverse events were coded using the Medical Dictionary for Regulatory Activities and summarised for each cohort by event severity and causality, as defined by the investigator.

Analysis was performed using SAS® software, version 9.4 (SAS Institute Inc., Cary, NC, USA).

## Results

### Patient demography and baseline characteristics

The DRAKO study enrolled 750 patients – 507 treatment-naive and 241 non–treatment-naive. Two patients were included in the non–treatment-naive cohort 12-month analysis prior to site confirmation that patients were treatment-naive (patients excluded from safety population and subsequent analysis). For the PPW populations, this equated to *N* = 388 and *N* = 326 for the treatment-naive cohort, and *N* = 169 and *N* = 135 for the non–treatment-naive cohort, at Months 12 and 24, respectively (Supplementary Figure 1). Comparable demographics and baseline measures were reported for each cohort population at Months 12 and 24 (Supplementary Table 1).

The non–treatment-naive patient cohort was older than the treatment-naive cohort (mean age of 64.5 years and 62.8 years, respectively). Patients in both cohorts were mostly male (approximately 60%) and white (77.6% treatment-naive and 63.7% non-treatment-naive). Almost 90% of patients in both cohorts were diagnosed with type 2 diabetes, and fellow eye involvement was higher in the non–treatment-naive cohort (61.5% vs 52.5%). Patients in the treatment-naive cohort had superior baseline BCVA (BCVA ≥ 70 letters, 64.4% treatment-naive and 59.3% non–treatment-naive), whereas patients in the non–treatment-naive cohort had lower baseline CST (CST < 400 µm, 15.4% treatment-naive and 37.8% non–treatment-naive). Almost 90% of patients in the non–treatment-naive cohort were previously treated with ranibizumab (Supplementary Table 1).

### Effect of treatment on functional and anatomical outcomes

For the treatment-naive cohort at Month 24, a marginal improvement from baseline in BCVA of 0.7 (12.7) letters was reported and CST continued to decrease in Year 2, with a change from baseline of −123.3 (104.3) µm (Table 1). For the non–treatment-naive cohort, BCVA remained stable at both Months 12 and 24 (0.2 and −0.3 letters, respectively), and a mean (SD) improvement in CST was observed (–79.1 [137.6] µm and –91.6 [132.9] µm) at Months 12 and 24, respectively.

**Table 1:** Mean change from baseline in best corrected visual acuity (BCVA) and central subfield thickness (CST) outcomes at Month 12 (M12) and Month 24 (M24) for the treatment-naïve and non-treatment-naïve patient cohorts.

			Treatment-naïve		Non-treatment-naïve	
			Baseline	Change from baseline	Baseline	Change from baseline
BCVA (Letters)	M12 (*N* = 388)	Mean (SD)	71.4 (12.0)	2.5 (12.2)	68.8 (13.7)	0.2 (10.2)
		95% CI	-	1.3, 3.8	-	−1.5, 1.8
		*n*	375	353	166	153
	M24 (*N* = 326)	Mean (SD)	71.5 (12.4)	0.7 (12.7)	69.5 (12.8)	−0.3 (13.0)
		95% CI	-	−0.7, 2.1	-	−2.5, 1.9
		*n*	326	326	135	135
CST (µm)	M12 (*N* = 169)	Mean (SD)	448.7 (88.7)	−119.1 (116.4)	419.3 (121.0)	−79.1 (137.6)
		95% CI	-	−130.7, −107.4	-	−100.2, −58.0
		*n*	388	386	169	166
	M24 (*N* = 135)	Mean (SD)	447.6 (77.3)	−123.3 (104.3)	422.5 (117.8)	−91.6 (132.9)
		95% CI	-	−134.7, −112.0	-	−114.2, −69.0
		*n*	326	326	135	135

95% confidence intervals (CI) are stated.

At Month 24, 16.6% of treatment-naive patients reported a ≥ 10 letter gain from baseline, compared to 20.1% at Month 12 (Table 2). At Month 24, more patients had lost ≥ 5, ≥ 10 or ≥ 15 letters compared to Month 12. A comparable number of injections was reported for the letter gain/loss subgroups and study mean in Year 1 (range: 6.4–6.7 injections). In Year 2, patients with ≥ 15 letter gain had a mean of 0.8 more injections than those with comparable letter loss.

**Table 2:** Proportion (%) of treatment-naïve and non-treatment-naïve patients experiencing greater than 5, 10 or 15 best corrected visual acuity (BCVA) letter gains or losses at Month 12 (M12) and Month 24 (M24).

			Treatment-naïve				Non-treatment-naïve			
Letter Change			Proportion of patients *n* (%)	Number of injections Mean (SD)	*r*	*p*-value	Proportion of patients *n* (%)	Number of injections Mean (SD)	*r*	*p*-value
M12	All Patients		388 (100)	-	-	-	169 (100)	-	-	-
	Patients Receiving IVT-AFL		388 (100)	6.4 (2.1)	-	-	169 (100)	6.0 (2.4)	-	-
	Gain	≥ 5	156 (40.2)	6.4 (1.9)	0.19	0.02	51 (30.2)	6.6 (2.4)	0.00	0.97
		≥ 10	78 (20.1)	6.5 (2.1)	0.28	0.01	25 (14.8)	6.8 (2.6)	−0.02	0.93
		≥ 15	34 (8.8)	6.8 (2.2)	0.44	0.01	8 (4.7)	6.9 (2.2)	−0.05	0.91
	Loss	≥ 5	64 (16.5)	6.4 (2.1)	0.01	0.94	42 (24.9)	5.6 (2.4)	−0.07	0.65
		≥ 10	38 (9.8)	6.5 (2.0)	0.05	0.77	24 (14.2)	5.7 (2.1)	−0.15	0.49
		≥ 15	23 (5.9)	6.7 (2.2)	0.20	0.36	9 (5.3)	6.1 (2.6)	−0.15	0.71
M24	All Patients		326 (100)	-	-	-	135 (100)	-	-	-
	Patients Receiving IVT-AFL		241 (73.9)	3.7 (2.3)	-	-	106 (78.5)	4.3 (2.5)	-	-
	Gain	≥ 5	124 (38.0)	3.6 (2.1)	0.08	0.42	43 (31.9)	4.5 (2.5)	−0.04	0.83
		≥ 10	54 (16.6)	3.7 (2.3)	0.18	0.26	20 (14.8)	4.7 (2.0)	−0.18	0.47
		≥ 15	27 (8.3)	4.1 (2.4)	0.02	0.92	9 (6.7)	4.4 (2.0)	−0.30	0.51
	Loss	≥ 5	85 (26.1)	3.7 (2.3)	0.03	0.81	39 (28.9)	4.7 (2.6)	0.14	0.47
		≥ 10	50 (15.3)	3.6 (2.2)	0.01	0.96	25 (18.5)	4.2 (2.1)	0.09	0.73
		≥ 15	24 (7.4)	3.3 (1.8)	−0.18	0.45	17 (12.6)	4.2 (2.2)	0.09	0.78

The mean (SD) number of intravitreal aflibercept injections for each subgroup is provided based on those patients receiving intravitreal aflibercept injections in each year. The correlation between letter gain or loss and the number of intravitreal aflibercept is represented by correlation coefficient (r) and associated *p*-value. The ‘All Patients’ cohort size and mean number of injections administered in year 1 (M12) and year 2 (M24) for each cohort have been included for comparison.

For the non–treatment-naive patients, 30.2% reported a ≥ 5 letter gain compared to 24.9% who experienced the same letter loss at Month 12 (Table 2). Comparable proportions of non–treatment-naive patients had ≥ 10 or ≥ 15 letter gains or losses at Month 12. At Month 24, a similar percentage of patients had ≥ 5 and ≥ 10 letter gains or losses; however, twice as many patients experienced a letter loss of ≥ 15 compared to those with the equivalent letter gains. In Year 1, patients experiencing a reduction in letters received approximately one less injection than those experiencing letter gains. In general, the correlation between the number of injections and letter gains or losses was low across the subgroups.

**Fig. 1:**
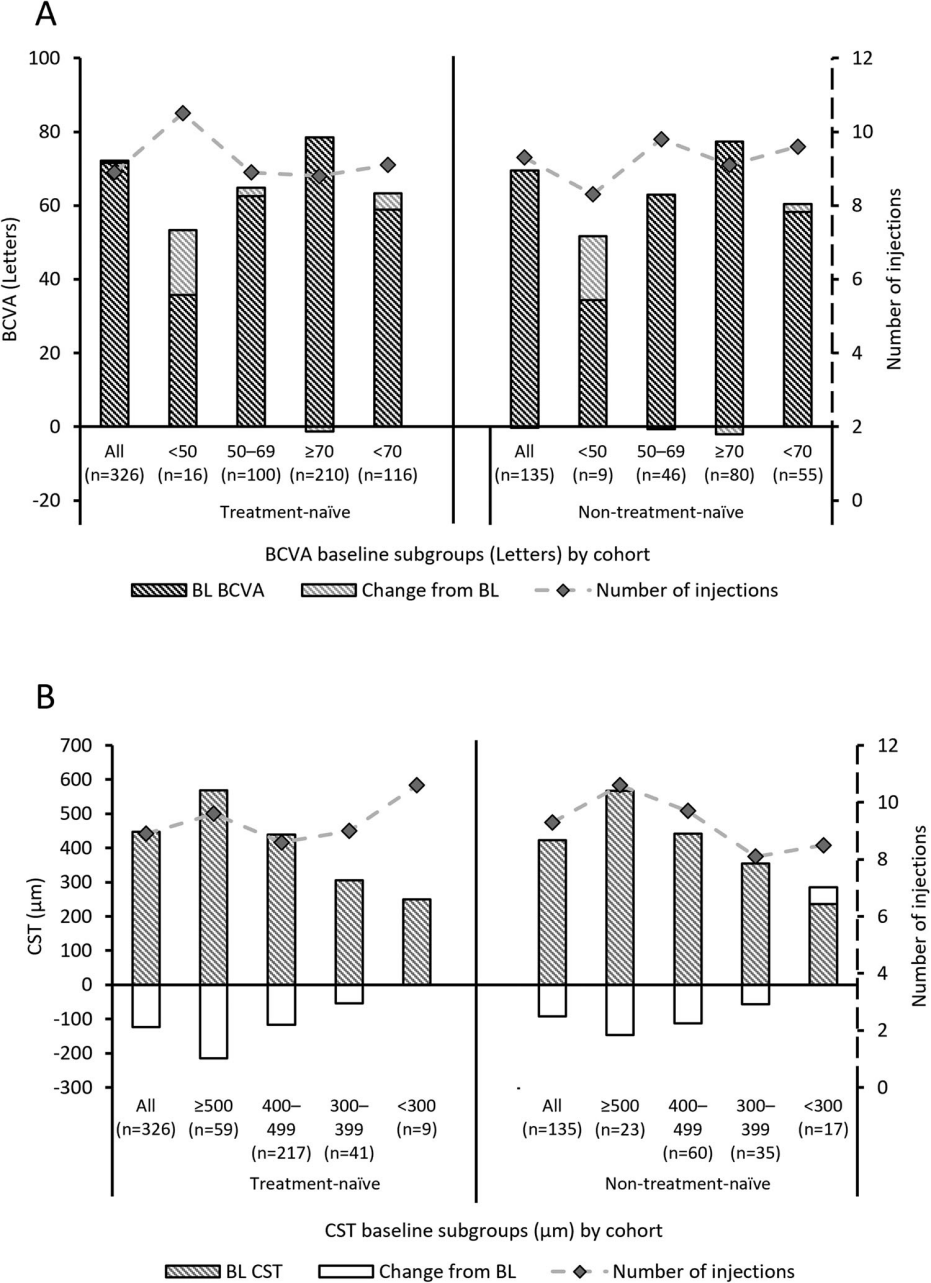
Mean change from baseline (BL) in best-corrected visual acuity (BCVA) and central subfield thickness (CST) outcomes at Month 24 for the treatment-naïve and non-treatment-naïve cohorts by mean baseline subgroup. The mean number of injections over the 2-year follow-up period is plotted as a dashed line on a secondary y-axis for each subgroup. **A** BCVA outcome by BCVA baseline subgroup for the treatment-naive and non-treatment-naïve cohorts. **B** CST outcome by CST baseline subgroup for the treatment-naïve cohort and non-treatment-naïve cohorts. BCVA Best-corrected visual acuity, BL Baseline, CST Central subfield thickness, n Number of patients per group.

BCVA and CST outcomes at Month 24 were affected by baseline measures for both cohorts (Fig. 1). Those patients with inferior baseline measures for BCVA ( < 50 letter subgroup) and CST ( ≥ 500 µm) experienced the greatest improvements, with mean change from baseline at Month 24 in BCVA of 17.6 letters and 17.3 letters and in CST of –214.8 µm and –146.7 µm for the treatment-naive and non–treatment-naive cohorts, respectively. Patients reporting baseline BCVA < 70 experienced letter gains above the study mean at Month 24 (4.4 letters and 2.2 letters in the treatment-naive and non–treatment-naive cohorts, respectively).

No trend was observed when BCVA and CST Month 24 outcomes were assessed based on the number of injections administered in Year 2, either by increasing injection number or baseline measure for either cohort (Supplementary Tables 2 and 3).

### Effect of treatment on vision-related QoL

**Fig. 2:**
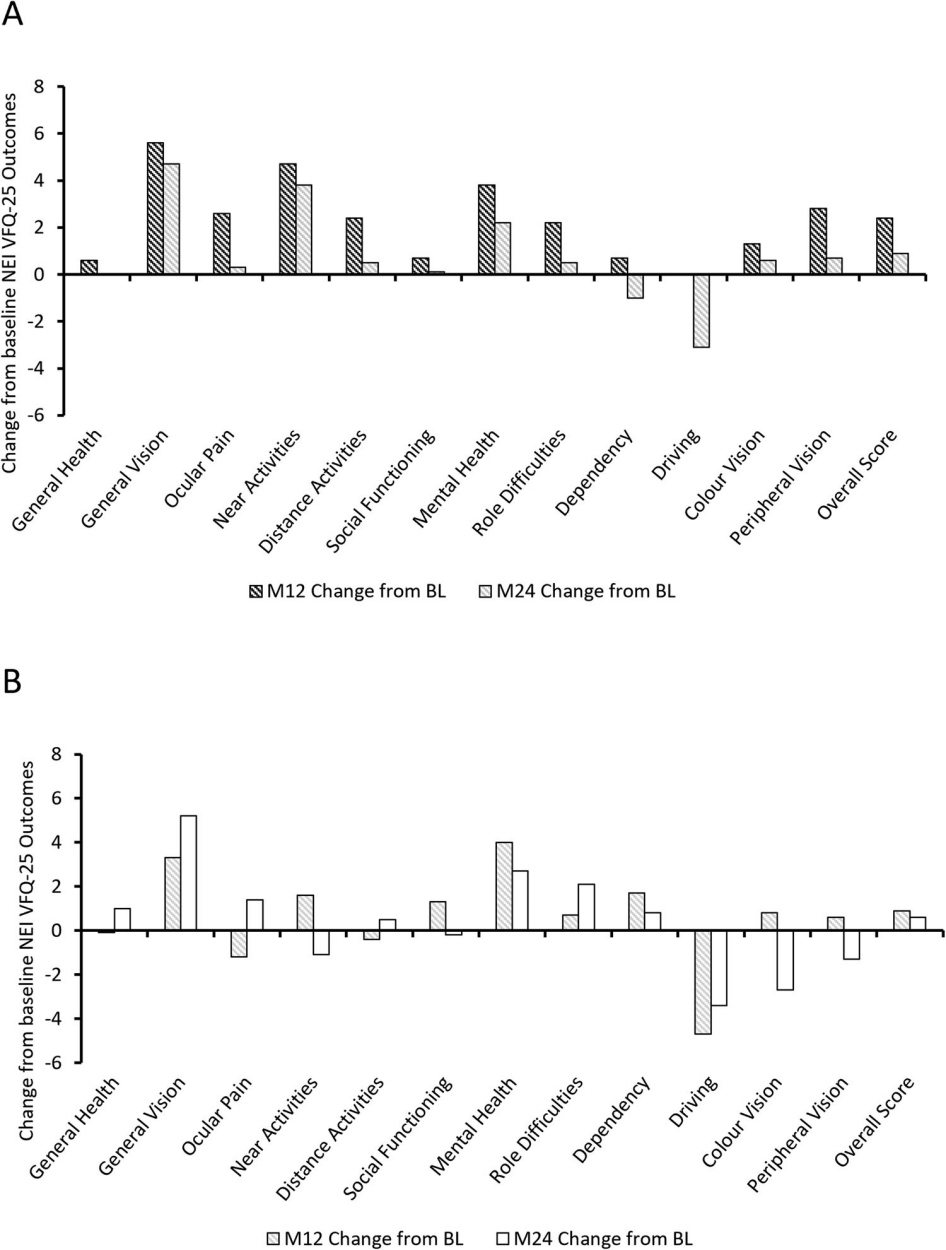
Mean change from baseline in NEI VFQ-25 metrics at Month 12 (M12) and Month 24 (M24). Changes in the NEI VFQ-25 National Eye Institute Visual Function Questionnaire 25 item version for sub-scales and the overall score are presented for **A** Treatment-naive cohorts, **B** Non-treatment-naive cohorts.

High vision-related QoL was reported by patients using the NEI VFQ-25 instrument, with overall baseline scores of 80.4 and 77.3 reported for the 24-month treatment-naive and non–treatment-naive cohorts, respectively (Supplementary Table 4). Comparable trends in baseline sub-scale scores were observed between the cohorts, with general health and general vision reported as the lowest sub-scale scores for both cohorts (47.2 versus 47.0 for general health; 47.7 versus 45.0 for general vision, treatment-naive cohort and non–treatment-naive, respectively). In line with the functional outcomes at Months 12 and 24, largest improvements in the mean change from baseline were observed at Month 12 for both cohorts. Augmentation from baseline in sub-scale scores at Month 24 was reported for general vision (4.7) and near activities (3.8) for the treatment-naive cohort, with the greatest loss reported in the driving sub-scale (–3.1). General vision (5.2) and mental health (2.7) were reported as the largest gains from baseline in the non–treatment-naive cohort at Month 24, with driving score (–3.4) reporting the greatest loss (Fig. 2).

### Effect of treatment DMO monitoring assessments

DR was mostly measured using the English grading system (83.4% for treatment-naive and 87.5% for non–treatment-naive cohorts). At baseline, most treatment-naive patients reported background or pre-proliferative retinopathy (R1 = 38.7%; R2 = 32.1%) or mild or observable retinopathy (R1 = 45.7%; R2 = 41.3%) based on the English and Scottish scales, respectively (Table 3). At Month 12, DR grading was predominantly stable for the English scale, with a 7% reduction in the proportion of patients reported with pre-proliferative retinopathy (R2), although missing values may have contributed to this finding. A more noticeable improvement in DR was observed in patients assessed using the Scottish scale where classification of mild retinopathy (R1) or better increased by 6 (13%) from a baseline of 21 patients. Month 24 outcomes were comparable to Month 12, demonstrating stability of the DR grade post-treatment. Similar trends were observed for the non–treatment-naive cohort, at Month 12 and Month 24.

**Table 3:** Diabetic retinopathy outcomes at Month 12 (M12) and Month 24 (M24) for the treatment-naïve and non-treatment-naïve cohort.

		Treatment-naïve				Non-treatment-naïve			
		M12 (*N* = 388)		M24 (*N* = 326)		M12 (*N* = 169)		M24 (*N* = 135)	
		BL *n* (%)	M12 *n* (%)	BL *n* (%)	M24 *n* (%)	BL *n* (%)	M12 *n* (%)	BL *n* (%)	M24 *n* (%)
**English**	**R0**	3 (1.2)	-	3 (1.2)	4 (1.7)	1 (0.9)	2 (1.9)	1 (0.9)	3 (2.8)
	**R1**	94 (38.7)	98 (40.3)	94 (39.0)	95 (39.4)	37 (34.9)	33 (31.1)	37 (34.9)	37 (34.9)
	**R2**	78 (32.1)	61 (25.1)	78 (32.4)	50 (20.7)	35 (33.0)	30 (28.3)	35 (33.0)	27 (25.5)
	**R3**	41 (16.9)	38 (15.6)	41 (17.0)	46 (19.1)	18 (17.0)	20 (18.9)	18 (17.0)	17 (16.0)
	**Missing**	27 (11.1)	46 (18.9)	25 (10.4)	46 (19.1)	15 (14.2)	21 (19.8)	15 (14.2)	22 (20.8)
**Scottish**	**R0**	-	1 (2.2)	-	-	-	-	-	-
	**R1**	21 (45.7)	26 (56.5)	21 (43.8)	28 (58.3)	4 (30.8)	8 (61.5)	4 (28.6)	6 (42.9)
	**R2**	19 (41.3)	10 (21.7)	19 (39.6)	10 (20.8)	5 (38.5)	2 (15.4)	5 (35.7)	3 (21.4)
	**R3**	3 (6.5)	5 (10.9)	3 (6.3)	7 (14.6)	4 (30.8)	2 (15.4)	4 (28.6)	4 (28.6)
	**R4**	-	1 (2.2)	-	-	-	1 (7.7)	-	-
	**Missing**	3 (6.5)	3 (6.5)	5 (10.4)	3 (6.3)	-	-	1 (7.1)	1 (7.1)

Outcomes are provided based on the English National Screening Committee (English) or Scottish Diabetic Retinopathy Grading Scheme (Scottish) classifications as appropriate.

English scale, *R0* No visible retinopathy, *R1* Background retinopathy, *R2* Pre-proliferative retinopathy, *R3* Proliferative retinopathy.

Scottish; *R0* No visible retinopathy, *R1* Mild retinopathy, *R2* Observable retinopathy, *R3* Referable retinopathy, *R4* Proliferative retinopathy.

Mean baseline HbA_1c_ measures indicated poor levels of control for both treatment-naive (66.1 mmol/mol) and non–treatment-naive (66.8 mmol/mol) cohorts (Supplementary Table 5). Mean changes from baseline at Months 12 and 24 were small for both treatment-naive patients (−2.9 mmol/mol and + 0.4 mmol/mol) and the non–treatment-naive patients (+ 2.7 mmol/mol and –0.7 mmol/mol), respectively.

### Safety

Over the 2-year follow-up period, 950 adverse events (AEs) were reported (713 in the treatment-naive and 237 in the non–treatment-naive cohorts), of which 40.7% were serious AEs (Supplementary Table 6). Most AEs were treatment emergent (99.8%) and were classified as non-eye disorders (80.5%). A small proportion of AEs were determined to have a reasonable causal relationship with the injection procedure (6.0%) or the treatment (3.7%). A review of key events with a causal relationship with the IVT-AFL procedure determined cataracts as the most frequent AE: 1.5% across the study and affecting 13 (1.7%) patients (Supplementary Table 7). Occurrence of key causal related events was low, with endophthalmitis reported in 2 (0.3%) patients, intraocular pressure increase reported in 4 (0.5%) patients and injection-site pain reported in 9 (1.2%) patients, with most causal AEs classified as non-serious AEs. The safety profile for the study was comparable with other IVT-AFL published studies, and no new safety concerns were identified.

## Discussion

The DRAKO study results report that baseline VA was maintained at 24 months, with BCVA remaining > 70 letters and CST continuing to decrease in Year 2. For patients who switched to IVT-AFL treatment from other anti-VEGF agents, almost 90% of whom previously received ranibizumab, VA remained steady throughout the 2-year follow-up period, with 24-month BCVA at almost 70 letters and minor fluctuations observed at Months 12 and 24. Although superior baseline anatomical measures were reported for the non–treatment-naive patients, improvement from baseline was not as pronounced as that observed in the treatment-naive cohort, with similar CST outcomes at 24 months in both cohorts. As previously reported at 12 months for the treatment-naive cohort [10], the baseline BCVA for DRAKO patients was considerably higher than that seen in RCTs [11, 12], which may have introduced a ‘ceiling’ effect that reduced the potential for larger visual acuity gains. This is supported by a mean gain of + 7.3 letters at 12 months for Cohort 1 patients with a baseline BCVA < 70 letters [10]. Additionally, several RCTs [9, 13] have shown that most VA gains are experienced in the first year of anti-VEGF treatment. In the pivotal VIVID and VISTA [9] RCTs, between 52 and 100 weeks, the 8-weekly treatment cohorts reported a loss of 1.3 letters and a small 0.4 letter gain, respectively. In Protocol T [13], a 0.5 letter loss was reported from 12 to 24 months for the IVT-AFL cohort. It is therefore not surprising that DRAKO observed small reductions of 1.8 and 0.5 letters from 12 to 24 months for the treatment-naive and non–treatment-naive cohorts, respectively.

Another significant differentiator between real-world evidence studies (RWE) and RCTs is the absence of a regimented dosing requirement in RWE studies. In DRAKO, 12-month results demonstrated that the mean number of injections in Year 1 for the treatment-naive cohort and non–treatment-naive cohort was 6.4 and 6.0, respectively, compared to the recommended 8 to 9 injections referenced in the IVT-AFL SmPC posology for DMO treatment [7]. In Year 2, DRAKO reports that the treatment-naive cohort and non–treatment-naive cohort patients received a mean of 3.7 and 4.3 injections, respectively, with the majority of the treatment-naive cohort receiving two injections or less and a quarter receiving none. For the non–treatment naive cohort, treatment numbers were slightly higher, with the majority of patients receiving three or less injections in Year 2 and a fifth receiving none. As reasons for treatment choices were not collected, it was not possible to determine why significant numbers of patients did not receive additional treatments and overall injection numbers were lower than the SmPC recommendation, even when allowing for the implementation of a ‘treat-and-extend’ posology, whereby treatment intervals may have been extended based on visual and/or anatomical outcomes. Possible contributing factors could include NHS capacity constraints, clinical prioritisation or patient influences. Interestingly, even lower injection numbers were recently reported in Japanese clinical practice, suggesting a trend in real-world settings [14]. Based on these findings, further improvements in visual outcomes may have been possible, although VA was maintained at a high baseline level.

Evaluation of treatment patterns across the study indicated that treatment was relatively standardised across the UK, with little variation in the number of IVT-AFL injections administered based on baseline measures. Rather, baseline measures were a better indicator of Month 12 and Month 24 outcomes than the number of injections administered. Treatment patterns were not indicative of a personalised medicine approach, as baseline factors did not appear to influence injection numbers, nor was there a difference in injection numbers between those who experienced a gain of 15 letters or more and those who lost 15 letters or more. However, as overall patient outcomes were very similar irrespective of the numbers of injections, it is possible that injections were administered to reach a clinical goal and reduced thereafter, whereas administering more injections, especially in Year 1 of treatment, may have delivered greater vision improvements [9, 12].

The high baseline QoL scores reported across both cohorts were maintained, with modest increases reported at 12 and 24 months, and results closely reflecting the BCVA outcomes, confirming previously reported findings that QoL outcomes are associated with VA outcomes [9]. Additionally, DR outcomes were consistent with the overall study outcomes, where little variation in DR grade was reported. The Scottish grading outcomes showed greater improvement, with significant movement from R2 to R1. This is likely to reflect the increased granularity of the Scottish scale, where mild (R1) and moderate background DR (R2) are graded separately, whereas background DR is combined in the English scale (R1). Interestingly, the Scottish scale grading also reported a significantly smaller proportion of patients with referable/pre-proliferative or proliferative retinopathy.

It was previously reported that DRAKO patients in the anti-VEGF treatment-naive cohort demonstrated poor baseline glycaemic control, with a mean (SD) HbA_1c_ of 66.1 (20.5) mmol/mol [10]. During the 2-year follow-up period, little change was observed in mean HbA_1c_ levels for either cohort, with minor fluctuations resulting in 24-month changes from a baseline of 0.4 and −0.7 mmol/mol for the treatment-naive cohort and non-treatment-naive cohort, respectively. The International Diabetes Federation recommends that a general target for glucose control in type 2 diabetes should be less than 53 mmol/mol, although a target of 58–64 mmol/mol may be appropriate in patients with more severe conditions; values above 64 mmol/mol are generally unacceptable [15]. Based on such recommendations, DRAKO participants may have benefited from a more proactive focus on glycaemic control to improve patient outcomes during the study and longer term.

The study has some limitations often inherent in observational studies, such as inconsistent treatment administration and non-defined functional eligibility metrics. However, the prospective study design and wide range of contributing sites enabled treatment effects to be monitored across a diverse, UK-representative population.

In conclusion, Year 2 results confirm the earlier 12-month findings reported for the treatment-naive cohort [10] that IVT-AFL is an effective treatment for DMO in real-world UK clinical practice. The high baseline VA and QoL scores were maintained over the 2 years and anatomical outcomes continued to improve in the second year. Although DRAKO has shown that patients with DMO in the UK are identified and treated with a high baseline VA that is being maintained over 2 years, the low overall injection numbers and sub-optimal glycaemic control may provide important opportunities to further improve patient care in the future.

## Summary

### What was known before

- The effectiveness of intravitreal aflibercept (IVT-AFL) for the treatment of patients with diabetic macular oedema (DMO) has been demonstrated in several pivotal clinical trials (VIVID and VISTA) and non-UK-focused observational studies (APOLLON), although such investigations primarily focused on patients with baseline visual acuity of < 73 letters.
- Retrospective registry-based studies of anti-vascular endothelial growth factor (VEGF) treatments have reported lower injection frequency and functional gains than randomised clinical trials.
- The scope for improvement of functional and anatomical parameters in response to anti-VEGF treatment is closely associated with baseline values.

### What this study adds

- DRAKO is the first prospective observational study to evaluate IVT-AFL treatment of patients with DMO across the UK; Year 1 results for the anti-VEGF treatment-naïve cohort demonstrated the effectiveness of this treatment to maintain or improve patient outcomes across a diverse range of local standard of care protocols, despite often observing undertreatment compared to locally defined treatment plans.
- DRAKO Year 2 results confirm that standard of care IVT-AFL treatment of patients with DMO in the UK remains effective, maintaining visual acuity at high baseline levels and further improving anatomic outcomes, despite continued poor glycaemic control among patients and undertreatment compared to the SmPC recommendations.
- DRAKO indicates the effectiveness of the diabetic retinopathy screening programme in the UK, where patients with DMO are being identified and treated at a high level of visual acuity, thereby preserving patient vision.

## Supplementary information

Supplementary Figure 1

Supplementary Tables 1-7

## Data Availability

Availability of the data underlying this publication will be determined later according to Bayer’s commitment to the EFPIA/PhRMA “Principles for responsible clinical trial data sharing.” This pertains to scope, time point and process of data access. As such, Bayer commits to sharing upon request from qualified scientific and medical researchers patient-level clinical trial data, study-level clinical trial data, and protocols from clinical trials in patients for medicines and indications approved in the United States (US) and European Union (EU) as necessary for conducting legitimate research. This applies to data on new medicines and indications that have been approved by the EU and US regulatory agencies on or after January 01, 2014. Interested researchers can use www.clinicalstudydatarequest.com to request access to anonymized patient-level data and supporting documents from clinical studies to conduct further research that can help advance medical science or improve patient care. Information on the Bayer criteria for listing studies and other relevant information is provided in the study sponsors section of the portal. Data access will be granted to anonymized patient-level data, protocols and clinical study reports after approval by an independent scientific review panel. Bayer is not involved in the decisions made by the independent review panel. Bayer will take all necessary measures to ensure that patient privacy is safeguarded.
