# Supplementary material for: Evaluation of care with intravitreal aflibercept treatment for UK patients with diabetic macular oedema: DRAKO study 24-month real-world outcomes
Source: Eye (Lond). 2023 Mar 20;37(13):2753–60. doi: 10.1038/s41433-023-02409-y (PMC10482829; doi:10.1038/s41433-023-02409-y)
Supplement: Supplementary file 2 — Supplementary Tables 1-7 [file 41433_2023_2409_MOESM2_ESM.docx]

**Supplementary Tables**

**Supplementary Table 1.** Baseline demographics for treatment-naïve and non-treatment-naïve patient cohorts for Month 12 (M12) and Month 24 (M24) cohort populations.

|  | **Treatment-naïve** | | **Non-treatment-naïve** | |
| --- | --- | --- | --- | --- |
|  | **M12 (n=388)** | **M24 (n=326)** | **M12 (n=169)** | **M24 (n=135)** |
| **Age, years** | | | | |
| **All, mean (SD)** | 62.9 (11.6) | 62.8 (11.2) | 64.6 (10.8) | 64.5 (10.7) |
| **Age group, n (%)** | | | | |
| **18 – 35** | 9 (2.3) | 6 (1.8) | 2 (1.2) | 1 (0.7) |
| **36 – 50** | 40 (10.3) | 33 (10.1) | 14 (8.3) | 11 (8.1) |
| **51 – 65** | 177 (45.6) | 150 (46.0) | 63 (37.3) | 51 (37.8) |
| **>65** | 162 (41.8) | 137 (42.0) | 90 (53.3) | 72 (53.3) |
| **Sex, n (%)** | | | | |
| **Male** | 245 (63.1) | 206 (63.2) | 101 (59.8) | 80 (59.3) |
| **Female** | 143 (36.9) | 120 (36.8) | 68 (40.2) | 55 (40.7) |
| **Race or ethnicity, n (%)** | | | | |
| **White** | 298 (76.8) | 253 (77.6) | 109 (64.5) | 86 (63.7) |
| **Asian** | 38 (9.8) | 35 (10.7) | 34 (20.1) | 27 (20.0) |
| **Black** | 29 (7.5) | 21 (6.4) | 13 (7.7) | 10 (7.4) |
| **Mixed** | 4 (1.0) | 3 (0.9) | - | - |
| **Hispanic** | 2 (0.5) | 2 (0.6) | 1 (0.6) | 1 (0.7) |
| **Unknown** | 14 (3.6) | 8 (2.5) | 8 (4.7) | 7 (5.2) |
| **Other** | 3 (0.9) | 4 (1.2) | 4 (2.4) | 4 (2.8) |
| **Diabetes type, n (%)** | | | | |
| **1** | 55 (14.2) | 41 (12.6) | 18 (10.7) | 15 (11.1) |
| **2** | 333 (85.8) | 285 (87.4) | 151 (89.3) | 120 (88.9) |
| **Time to baseline, mean (SD) Years** | | | | |
| **Diabetes diagnosis to baseline** | 16.0 (10.5) | 15.9 (10.3) | 16.5 (9.1) | 16.7 (9.1) |
| **DMO diagnosis to baseline** | 1.3 (2.4) | 1.4 (2.5) | 3.4 (2.6) | 3.5 (2.7) |
| **Fellow eye involvement, n (%)** | | | | |
| **Yes** | 209 (53.9) | 171 (52.5) | 111 (65.7) | 83 (61.5) |
| **No** | 179 (46.1) | 155 (47.5) | 58 (34.3) | 52 (38.5) |
| **Baseline BCVA, n (%)** | | | | |
| **≤49** | 19 (4.9) | 16 (5.0) | 14 (8.3) | 9 (6.7) |
| **50 – 69** | 111 (28.6) | 100 (30.7) | 59 (34.9) | 46 (34.1) |
| **≥70** | 245 (63.1) | 210 (64.4) | 93 (55.0) | 80 (59.3) |
| **<70** | 130 (33.5) | 116 (35.6) | 73 (43.2) | 55 (40.7) |
| **Baseline CST, n (%)** | | | | |
| **<300** | 13 (3.4) | 9 (2.8) | 22 (13.0) | 16 (11.9) |
| **300 – 399** | 51 (13.1) | 41 (12.6) | 47 (27.8) | 35 (25.9) |
| **400 – 499** | 251 (64.7) | 217 (66.6) | 65 (38.5) | 60 (44.4) |
| **≥500** | 73 (18.8) | 59 (18.1) | 35 (20.7) | 24 (17.8) |
| **Prior anti-VEGF, n (%)** | | | | |
| **Ranibizumab only** | - | - | 140 (82.8) | 106 (78.5) |
| **Bevacizumab only** | - | - | 9 (5.3) | 8 (5.9) |
| **Ranibizumab and Bevacizumab** | - | - | 12 (7.1) | 13 (9.6) |
| **Unknown** | - | - | 8 (4.7) | 8 (5.9) |

**Supplementary Table 2**. The number of injections administered in year 2 of study follow-up and the associated mean (SD) baseline and change from baseline values for best corrected visual acuity (BCVA) and central subfield thickness (CST) in the treatment-naïve cohort.

| **Treatment-naïve cohort** | | | | | | |
| --- | --- | --- | --- | --- | --- | --- |
| **Number of Injections Year 2** | **BCVA (Letters)** | | | **CST (µm)** | | |
|  | **Baseline Mean (SD)** | **Change from baseline**  **Mean (SD)** | **n** | **Baseline Mean (SD)** | **Change from baseline**  **Mean (SD)** | **n** |
| **1** | 71.3 (10.9) | 2.8 (12.0) | 39 | 450.1 (85.1) | -118.0 (128.0) | 39 |
| **2** | 73.2 (12.0) | -1.1 (10.7) | 52 | 443.6 (54.0) | -104.8 (85.2) | 52 |
| **3** | 71.8 (10.9) | 1.0 (14.7) | 36 | 460.4 (76.9) | -162.5 (103.3) | 36 |
| **4** | 70.8 (13.4) | 1.0 (11.3) | 42 | 436.8 (82.5) | -121.8 (101.7) | 42 |
| **5** | 71.1 (15.9) | -1.1 (20.1) | 31 | 446.4 (89.7) | -122.7 (104.6) | 31 |
| **6** | 70.2 (12.6) | 5.9 (5.8) | 15 | 510.7 (108.7) | -153.9 (130.2) | 15 |
| **7** | 75.8 (6.9) | 0.6 (11.7) | 11 | 445.6 (46.0) | -141.0 (79.7) | 11 |
| **8** | 59.1 (18.0) | 4.7 (14.1) | 7 | 423.3 (39.0) | -102.3 (103.8) | 7 |
| **9** | 68.5 (12.0) | 1.0 (5.7) | 2 | 566.5 (103.9) | -107.0 (132.9) | 2 |
| **10** | 77.0 (4.2) | 11.0 (11.3) | 2 | 355.5 (133.6) | -63.5 (85.6) | 2 |
| **11** | 75.0 (11.3) | -4.5 (6.4) | 2 | 335.0 (2.8) | -46.5 (48.8) | 2 |
| **12** | 67.5 (2.1) | -5.0 (12.7) | 2 | 550.5 (30.4) | -226.0 (39.6) | 2 |

**Supplementary Table 3.** The number of injections administered in year 2 of study follow-up and the associated mean (SD) baseline and change from baseline values for best corrected visual acuity (BCVA) and central subfield thickness (CST) in the non-treatment-naïve cohort.

| **Non-treatment-naïve cohort** | | | | | | |
| --- | --- | --- | --- | --- | --- | --- |
| **Number of Injections Year 2** | **BCVA (Letters)** | | | **CST (µm)** | | |
|  | **Baseline Mean (SD)** | **Change from baseline**  **Mean (SD)** | **n** | **Baseline Mean (SD)** | **Change from baseline**  **Mean (SD)** | **n** |
| **1** | 69.2 (12.2) | -2.4 (12.4) | 14 | 435.1 (225.2) | -127.9 (240.4) | 14 |
| **2** | 73.1 (8.2) | 1.0 (10.2) | 15 | 409.0 (90.2) | -99.5 (117.1) | 15 |
| **3** | 67.6 (15.9) | -1.8 (11.8) | 18 | 423.3 (98.0) | -52.6 (130.2) | 18 |
| **4** | 60.9 (13.2) | 6.8 (17.5) | 16 | 467.6 (110.1) | -83.9 (138.8) | 16 |
| **5** | 72.3 (7.2) | -0.6 (11.7) | 10 | 417.7 (78.1) | -46.6 (101.5) | 10 |
| **6** | 71.2 (8.8) | -8.0 (26.3) | 6 | 442.5 (75.5) | -178.5 (90.3) | 6 |
| **7** | 73.4 (9.0) | 2.3 (7.4) | 12 | 415.5 (126.6) | -91.4 (138.3) | 12 |
| **8** | 72.6 (12.6) | -3.1 (10.4) | 10 | 456.8 (100.7) | -75.6 (63.2) | 10 |
| **9** | 71.0 (6.6) | 2.7 (9.1) | 3 | 420.7 (143.8) | -113.3 (107.5) | 3 |
| **10** | 77.5 (6.4) | 0.0 (12.7) | 2 | 379.0 (32.5) | -28.0 (39.6) | 2 |

**Supplementary Table 4.** The mean baseline measure for each NEI VFQ-25 metric at Month 12 (M12) and Month 24 (M24) for Treatment-naïve and Non-treatment naïve populations

|  | **Treatment-naïve** | | | | **Non-treatment-naïve** | | | |
| --- | --- | --- | --- | --- | --- | --- | --- | --- |
| **Baseline NEI VFQ-25 Measures** | **M12 (n=388)** | | **M24 (n=326)** | | **M12 (n=169)** | | **M24 (n=135)** | |
|  | **Mean (SD)** | **n** | **Mean (SD)** | **n** | **Mean (SD)** | **n** | **Mean (SD)** | **n** |
| **General Health** | 46.9 (23.1) | 384 | 47.2 (23.0) | 325 | 46.5 (23.2) | 166 | 47.7 (22.8) | 132 |
| **General Vision** | 45.8 (16.4) | 373 | 46.3 (16.7) | 320 | 44.8 (17.2) | 160 | 45.0 (17.2) | 128 |
| **Ocular Pain** | 87.0 (18.4) | 384 | 87.1 (18.6) | 325 | 87.6 (17.1) | 167 | 86.4 (18.7) | 133 |
| **Near Activities** | 73.6 (23.7) | 383 | 74.0 (22.9) | 324 | 70.2 (24.4) | 167 | 72.9 (23.7) | 133 |
| **Distance Activities** | 82.8 (21.1) | 384 | 84.4 (20.2) | 325 | 79.4 (22.9) | 166 | 80.4 (23.3) | 132 |
| **Social Functioning** | 90.8 (18.9) | 383 | 91.1 (18.8) | 325 | 87.8 (22.5) | 164 | 88.7 (22.0) | 131 |
| **Mental Health** | 74.1 (25.4) | 384 | 74.5 (25.5) | 325 | 66.2 (29.7) | 167 | 69.3 (29.9) | 133 |
| **Role Difficulties** | 79.2 (26.0) | 383 | 78.8 (27.1) | 325 | 72.2 (30.4) | 167 | 73.7 (30.5) | 133 |
| **Dependency** | 88.6 (23.7) | 384 | 88.3 (24.4) | 325 | 80.1 (31.0) | 167 | 82.1 (29.5) | 133 |
| **Driving** | 82.9 (29.0) | 258 | 83.4 (29.1) | 215 | 75.2 (35.3) | 103 | 75.0 (36.5) | 83 |
| **Colour Vision** | 93.7 (16.2) | 380 | 94.0 (15.8) | 322 | 93.4 (18.6) | 163 | 95.0 (16.6) | 130 |
| **Peripheral Vision** | 85.3 (22.9) | 381 | 86.0 (23.0) | 322 | 84.0 (25.1) | 164 | 84.7 (25.7) | 131 |
| **Overall Score** | 80.0 (17.4) | 384 | 80.4 (17.2) | 325 | 76.1 (19.6) | 167 | 77.3 (19.7) | 133 |

**Supplementary Table 5.** Haemoglobin A1c (HbA_1c_) change from baseline outcomes at Month 12 (M12) and Month 24 (M24) for the treatment-naïve and non-treatment-naïve cohorts.

|  | **Treatment-naive** | | | | **Non-treatment-naive** | | | |
| --- | --- | --- | --- | --- | --- | --- | --- | --- |
| **HbA1c (mmol/mol)** | **M12 (n=388)** | | **M24 (n=326)** | | **M12 (n=165)** | | **M24 (n=135)** | |
|  | **Mean (SD)** | **n** | **Mean (SD)** | **n** | **Mean (SD)** | **n** | **Mean (SD)** | **n** |
| **Baseline** | 66.1  (20.5) | 194 | 65.8  (18.9) | 170 | 66.8  (18.0) | 60 | 66.3  (17.9) | 53 |
| **M12 or M24** | 65.7  (19.1) | 139 | 67.6  (21.2) | 95 | 63.6  (13.7) | 25 | 69.5  (20.1) | 29 |
| **Change from Baseline** | -2.9  (16.2) | 98 | 0.4  (17.2) | 71 | 2.7  (10.5) | 18 | -0.7  (13.6) | 18 |

**Supplementary Table 6.** Summary of treatment-emergent adverse events reported over the 2-year follow-up period (M24) for the treatment-naïve and non-treatment-naïve patient cohorts.

| **Category** | **Total Number of Events at M24** | **Treatment-naïve (N=507)** | | **Non-treatment-naïve (N=241)** | |
| --- | --- | --- | --- | --- | --- |
|  |  | **Events**  **n** | **Patients**  **n (%)** | **Events**  **n** | **Patients**  **n (%)** |
| Number of Adverse Events | 950 | 713 | 225 (44.4) | 237 | 88 (36.5) |
| Eye Disorders | 183 | 128 | 80 (15.8) | 55 | 35 (14.5) |
| Non-eye disorders | 767 | 585 | 199 (39.3) | 182 | 72 (29.9) |
| Of which are SAE | 387 | 297 | 123 (24.3) | 90 | 43 (17.8) |
| Eye Disorders | 29 | 22 | 16 (3.2) | 7 | 6 (2.5) |
| Non-eye disorders | 358 | 275 | 113 (22.3) | 83 | 38 (15.8) |
| Any Treatment Emergent AEs | 948 | 711 | 225 (44.4) | 237 | 88 (36.5) |
| Eye Disorders | 183 | 128 | 80 (15.8) | 55 | 35 (14.5) |
| Non-eye disorders | 765 | 583 | 199 (39.3) | 182 | 72 (29.9) |
| Any Non Treatment Emergent AEs | 2 | 2 | 1 (0.2) | - | - |
| Reasonable Causal Relationship between the AE and the Injection Procedure | 57 | 44 (6.2%) | 28 (5.5%) | 13 (5.5%) | 10 (4.1%) |
| Reasonable Causal Relationship between Intravitreal Aflibercept and AE | 35 | 26 (3.6%) | 16 (3.2%) | 9 (3.8%) | 6 (2.5%) |

**Supplementary Table 7.** Summary of key injection procedure causal associated adverse events by preferred term and severity reported over the 2-year follow-up period (M24) for the treatment-naïve and non-treatment-naïve patient cohorts.

|  | **Preferred term** | **Events,**  **n** | **Patients,**  **n (%)** | **SAE,**  **n** | **Causal relationship with procedure, n** | **Causal relationship with IVT-AFL, n** |
| --- | --- | --- | --- | --- | --- | --- |
| **Treatment-naïve**  **(N=507)** | Cataract | 10 | 9 (1.8) | 0 | 2 | 1 |
|  | Conjunctival haemorrhage | 2 | 2 (0.4) | 0 | 2 | 0 |
|  | Corneal abrasion | 5 | 5 (1.0) | 0 | 5 | 0 |
|  | Endophthalmitis | 2 | 2 (0.4) | 2 | 2 | 0 |
|  | Eye pain | 4 | 4 (0.8) | 0 | 2 | 0 |
|  | Eyelid pain | 1 | 1 (0.2) | 0 | 1 | 1 |
|  | Injection site pain | 5 | 5 (1.0) | 0 | 3 | 1 |
|  | Intraocular pressure increased | 5 | 4 (0.8) | 0 | 2 | 2 |
| **Non-treatment-naïve (N=241)** | Cataract | 4 | 4 (1.7) | 2 | 1 | 0 |
|  | Conjunctival haemorrhage | 1 | 1 (0.4) | 0 | 1 | 0 |
|  | Corneal abrasion | 4 | 4 (1.7) | 0 | 4 | 0 |
|  | Eye pain | 3 | 3 (1.2) | 0 | 1 | 0 |
|  | Injection site pain | 4 | 3 (1.2) | 0 | 3 | 3 |
